# Mental health after orchiectomy: Systematic review and strategic management

**DOI:** 10.1080/20905998.2025.2478771

**Published:** 2025-03-16

**Authors:** G. Shokaier, M. Gross, M. Cohen, A. Hussein

**Affiliations:** Department of Urology, Emek Medical Center, Affiliated to the Rappaport Faculty of Medicine, Haifa, Israel

**Keywords:** Orchiectomy, mental health, testosterone replacement therapy, body image, quality of life, psychological outcomes

## Abstract

Orchiectomy, the surgical removal of one or both testicles, is often a life-changing procedure. While it is a critical treatment step for conditions such as testicular cancer, advanced prostate cancer, severe unresolving infection, trauma and gender dysphoria, the associated psychological challenges remain underexplored. Depression, anxiety, body image concerns, and diminished quality of life are prevalent but insufficiently addressed. This review synthesizes available literature to quantify these psychological impacts, explore cultural and demographic influences, and present evidence-based management strategies. The review highlights the importance of pre-operative counselling, testosterone replacement therapy (TRT), cognitive behavioural therapy (CBT), and social support networks. Future research should focus on longitudinal assessments to better understand long-term mental health outcomes post-orchiectomy.

## Introduction

Orchiectomy is a well-established surgical intervention performed for oncological, endocrine, infectious, traumatic and gender-affirming indications. Despite its medical necessity, the psychosocial consequences of the procedure remain underappreciated. Hormonal depletion, self-image alterations, and cultural perceptions surrounding masculinity can significantly impact mental health outcomes. Studies indicate a heightened prevalence of depression, anxiety, and cognitive dysfunction among post-orchiectomy patients. This review aims to analyse the psychological effects, provide a synthesis of existing research, and suggest evidence-based management strategies to mitigate adverse outcomes.

## Materials and methods

A systematic review was conducted using PubMed, Scopus, and Web of Science to identify studies published between 2000 and 2024. Search terms included ‘orchiectomy,’ ‘psychological outcomes,’ ‘depression,’ ‘anxiety,’ ‘hormonal therapy,’ and ‘psychosocial interventions.’ Studies were included if they reported on psychological and quality-of-life outcomes in adult populations post-orchiectomy. Exclusion criteria included non-English publications.

**Figure 1.**
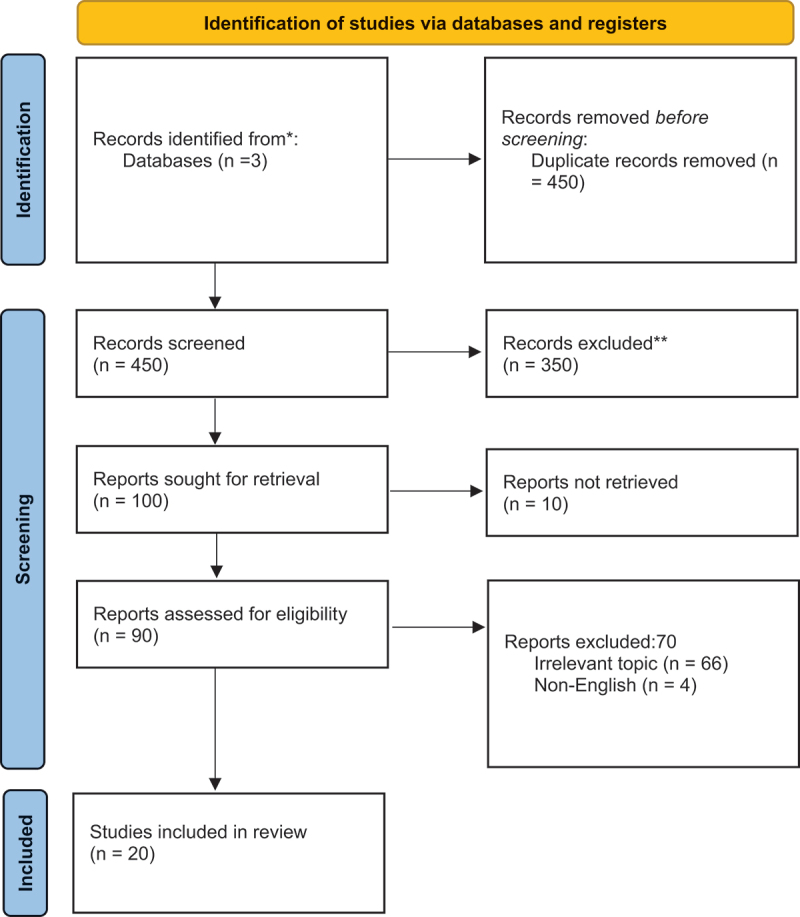
PRISMA flow diagram.

A total of 20 studies met the inclusion criteria and were included in the final analysis. A PRISMA flowchart (Figure 1) was generated to illustrate the study selection process.

## Results

### Psychological impacts of orchiectomy

#### Depression and anxiety

##### Testicular cancer

Testicular cancer (TC) is among the most curable malignancies, yet survivors often endure significant psychological distress, particularly depression and anxiety. Several studies have explored the mental health challenges faced by TC survivors, highlighting both risk factors and protective elements influencing psychological outcomes.

Dax et al. (2023) investigated the relationship between masculinity constructs and psychological distress in men with TC. Their findings revealed no significant differences in overall levels of depression and anxiety between TC survivors and healthy controls. However, subjective masculine stress and restrictive affectionate behaviour were uniquely associated with distress in the TC group. More specifically, restrictive emotionality, conflicts between work and family responsibilities, and subjective masculine stress were significantly correlated with psychological distress. These findings suggest that rigid adherence to traditional masculine norms, particularly those discouraging emotional expression and vulnerability, may exacerbate psychological distress in TC survivors. Conversely, optimistic action, a masculinity trait linked to perseverance and resilience, was associated with lower levels of depression and anxiety, suggesting that certain masculine constructs may be protective against psychological distress [1].

A large population-based study (Raphael et al., 2021) found that TC survivors had significantly higher rates of mental healthcare visits than age-, sex-, and geographically matched controls without a cancer history. The increased mental health service use emerged before orchiectomy and persisted throughout long-term survivorship, indicating that psychological distress is not confined to the acute treatment phase [2].

**Several key trends emerged from this study**

1. Primary care physicians were the most frequent providers of mental health services, emphasizing their critical role in addressing the psychological needs of TC survivors [2].
2. Patients with a history of mental health conditions and those undergoing upfront adjuvant therapy (e.g. chemotherapy, radiation, or retroperitoneal lymph node dissection) were at the highest risk for post-treatment mental health concerns [2]

These findings reinforce conclusions from Smith et al. (2021), whose systematic review reported that one in five TC survivor’s experiences significant anxiety, one in seven experiences distress, and one in ten experiences depression – rates that exceed those observed in the general population [3].

Beyond depression and anxiety, cognitive impairment (CI) is an emerging concern among testicular cancer (TC) survivors. Cognitive dysfunction has traditionally been linked to chemotherapy-related neurotoxicity; however, recent evidence suggests that CI may develop even before systemic treatment, potentially due to psychological distress and biological factors [4].

A study by Amidi et al. (2015) evaluating cognitive function in newly diagnosed and orchiectomized TC patients prior to systemic treatment found that 58% of patients exhibited cognitive impairment, a rate significantly higher than that observed in healthy controls. Compared to healthy men, TC patients performed significantly worse on six out of eleven neuropsychological tests, particularly in processing speed, attention, working memory, verbal learning, memory, and verbal fluency [4].

Biological and psychological factors were associated with cognitive dysfunction. Higher cortisol levels predicted poorer overall neuropsychological performance, suggesting a potential role of stress-induced neuroendocrine dysregulation. Additionally, cognitive complaints correlated with inflammatory markers (IL-6) and all psychological distress measures, further supporting the link between psychosocial stress and cognitive function in TC patients [4].

##### Prostate cancer patients on androgen deprivation therapy (ADT)

Prostate cancer (PCa) patients undergoing bilateral orchiectomy or Androgen Deprivation Therapy (ADT) often experience significant psychological distress, including depression, anxiety, and sleep disturbances. ADT, which involves medical or surgical castration, is a cornerstone treatment for advanced prostate cancer but has been linked to substantial mental health effects due to hormonal changes, psychosocial stress, and physical side effects [5].

A study by Louda et al. (2012) evaluated the psychosocial implications of bilateral orchiectomy in 89 prostate cancer patients. Key findings included:

1. Increased sleep disturbances post-orchiectomy, particularly during hospitalization, with some worsening post-discharge [5].
2. Greater demand for psychological support: 21% of patients sought psychiatric or psychological help post-orchiectomy, compared to only 10% pre-surgery [5].

These findings highlight the complex psychological impact of orchiectomy, showing increased mental distress (e.g. sleep disturbances, need for psychological support) [5]

A systematic review by Zhang et al. (2023) compared LHRH agonists and orchiectomy in prostate cancer patients, analysing therapeutic efficacy, adverse effects, and metabolic complications. The study found no significant difference in therapeutic outcomes, including PSA reduction, testosterone suppression, disease progression, or survival rates. However, psychological and metabolic complications were notable in both groups:

- Mental distress and pain:
  - LHRH agonists were associated with a higher risk of increased pain compared to orchiectomy, potentially contributing to chronic distress and decreased quality of life [6].
  - Emotional distress was comparable between surgical and medical castration, reinforcing the need for psychological support in PCa patients undergoing ADT [6].
- Metabolic effects influencing psychological well-being:
  - Both treatments led to metabolic disturbances, including lipid and glucose metabolism disorders [6].
  - LHRH agonists were associated with greater insulin resistance, while orchiectomy patients exhibited worse lipid metabolic effects [6].
  - The increase in body fat and metabolic changes can further contribute to depression, anxiety, and cognitive impairment, which are frequently observed in PCa patients undergoing ADT [6].
- Psychosocial and economic considerations:
  - Orchiectomy is a one-time procedure, ensuring high patient compliance and lower financial burden [6].
  - LHRH agonists require continuous medication use, which can contribute to treatment fatigue, financial strain, and medication non-adherence, all of which exacerbate mental distress [6].

##### Gender-Affirming orchiectomy

Orchiectomy is a key gender-affirming procedure for transfeminine individuals with gender dysphoria (GD). While historically associated with negative psychological effects, the context of gender-affirming surgery (GAS) differs significantly. For many individuals with GD, orchiectomy is a medically necessary procedure that alleviates distress associated with testosterone-driven secondary sex characteristics.Gender dysphoria is strongly linked to increased rates of depression, anxiety, and suicidal ideation [7]. Many transgender individuals experience significant distress due to testosterone-induced physical traits (e.g. facial hair, deep voice, body structure) that do not align with their gender identity. Psychological relief is often reported following gender-affirming medical or surgical interventions, including hormone therapy and orchiectomy.A systematic review by Wernick et al. (2019) examined the psychological effects of gender-affirming surgeries, including orchiectomy and vaginoplasty, in transfeminine individuals. The study found that gender-affirming surgery (GAS) significantly improves mental health outcomes, including [7]:

1. Reduction in gender dysphoria-related distress.
2. Lower rates of depression and anxiety post-orchiectomy.
3. Increased life satisfaction and psychological well-being.

Additionally, research suggests that early access to gender-affirming care, including orchiectomy, can reduce the risk of long-term psychological distress, self-harm, and suicidality in transgender individuals.

For some transfeminine individuals, orchiectomy is a critical mental health intervention. It provides permanent elimination of endogenous testosterone, allowing individuals to:

1. Discontinue or reduce reliance on estrogen and anti-androgens (e.g. spironolactone, cyproterone acetate), which may have undesirable side effects.
2. Experience a more congruent body image, reducing gender dysphoria.
3. Improve emotional stability and reduce mood fluctuations associated with hormone therapy.

A growing body of evidence suggests that psychological outcomes following orchiectomy in transgender individuals are overwhelmingly positive, particularly when performed in an affirming healthcare environment with proper preoperative counselling [7]

### Body image and sexual function

#### Testicular cancer patients

A study by Incrocci et al. (2002) assessed body image and sexual function in 166 testicular cancer patients treated with orchiectomy and radiotherapy, with 123 patients completing a follow-up questionnaire approximately 51 months post-treatment [8]. The findings suggest that sexual function remained largely preserved, with 92% of patients remaining sexually active, comparable to pre-treatment levels. Interest in sex, erectile function, and sexual satisfaction did not significantly differ from age-matched healthy controls. However, 17% of patients reported erectile difficulties, a percentage slightly higher than before treatment, though this was more strongly correlated with age rather than the orchiectomy itself. Additionally, 20% of patients reported a decrease in sexual pleasure and activity, while 32% felt their treatment negatively impacted their sex life.

Regarding body image, 52% of patients felt their body had changed post-treatment, but the degree of distress varied among individuals. Although testicular prostheses were available, only 12% of patients had received one, and many were unaware of the option at the time of orchiectomy. The absence of a prosthesis was associated with greater self-consciousness when undressing in public spaces such as gyms or swimming pools. However, the presence of a prosthesis did not significantly impact overall self-esteem or sexual confidence.

Fertility concerns were also noted, with 20% of patients expressing anxiety about fertility post-orchiectomy, a concern that was more pronounced among younger patients. Despite these concerns, sperm banking was not commonly discussed or pursued before treatment, highlighting a gap in pre-treatment counseling.

These findings emphasize that while orchiectomy may affect body image and sexual function, most patients adapt well, maintaining fulfilling sexual lives. Moreover, patient education regarding testicular prostheses, fertility preservation, and potential changes in body image should be improved to enhance long-term well-being in testicular cancer survivors [8].

A study conducted by Wortel et al. (2015) aimed to evaluate these effects prospectively in patients with stage I – II testicular seminoma. The study included 161 patients with a median age of 36 years who underwent orchiectomy and received adjuvant or prophylactic radiotherapy. Patients completed questionnaires assessing body image and sexual function before radiotherapy, three months after treatment, and six months post-treatment.

The study revealed that 61% of patients reported feeling that their body had changed post-orchiectomy, with 51% experiencing minor changes and 10% reporting moderate to severe changes in body image. Concerns about undressing in public spaces, such as at the gym or swimming pool, were present in 19% of patients, while 13% reported body image concerns during sexual activity. Younger patients were more likely to experience body image distress. Despite this, a significant portion of patients was not adequately informed about the option of receiving a testicular prosthesis. While 44% of patients were unaware of this possibility, only 9% had received a prosthesis, and most who did were satisfied with the outcome.

Sexual function remained stable in terms of sexual activity, with 91% of patients remaining sexually active throughout the study. However, erectile rigidity significantly declined after six months (*p* = 0.016), with the percentage of patients reporting fully rigid erections decreasing from 33% to 25%. Additionally, 23% of patients experienced a decrease in sexual interest, activity, and pleasure, and 45% of patients felt that their disease and treatment had negatively affected their sexual life.

The study also established a strong correlation between body image concerns and sexual dysfunction. Patients who experienced greater body image distress were more likely to report decreased sexual interest, pleasure, and erectile function. These findings highlight the importance of addressing psychological aspects of body image early in the treatment process.

Given the underutilization of testicular prostheses and the lack of pre-treatment discussions regarding body image concerns, the study emphasizes the need for better patient education, early psychosexual counselling, and proactive discussions about body image and sexual health. While most patients adapted to the changes, those with significant body image concerns were more likely to experience sexual dysfunction, underscoring the necessity of comprehensive post-treatment support. Ultimately, the study suggests that while orchiectomy and radiotherapy lead to short-term changes in body image and sexual function, addressing these issues early through proper counselling, education, and the availability of prosthetic options may significantly improve long-term psychological and sexual well-being in testicular cancer survivors [9].

#### Transgender women

Orchiectomy has long been associated with negative perceptions in certain medical contexts, particularly in cancer treatment. However, in gender-affirming care, orchiectomy can serve as a powerful, positive intervention for individuals seeking to align their bodies with their gender identity [10]. A study by Stelmar et al. (2024) highlights the endocrine, psychological, and sexual function benefits of gender-affirming bilateral orchiectomy (GABO) in transgender women and nonbinary individuals [10]. One of the primary benefits of orchiectomy in gender-affirming care is the reduction of gender dysphoria. Many transgender women and nonbinary individuals experience significant distress due to endogenous testosterone production, which drives undesired secondary sex characteristics. The study found that over 96% of patients reported a reduction in gender dysphoria after undergoing GABO, indicating its profound psychological relief. Additionally, patients who underwent GABO as a standalone procedure before vaginoplasty reported immediate improvements in body image, confidence, and comfort with their physical appearance [10]. Another significant advantage of orchiectomy is its impact on gender-affirming hormone therapy (GAHT). Following the removal of testosterone-producing organs, patients can significantly reduce or discontinue androgen blockers such as spironolactone, which often come with undesirable side effects like dizziness, fatigue, and electrolyte imbalances. The study found that 100% of patients discontinued spironolactone after surgery, and estradiol and progesterone dosages were also significantly reduced. This reduction in medication not only lowers health risks (such as cardiovascular complications associated with high-dose estradiol) but also improves overall well-being and stability in hormone levels. From a sexual health perspective, GABO has been associated with improvements in sexual function and satisfaction. The study reported that a majority of patients experienced increased sexual confidence and a greater sense of comfort in intimate settings post-surgery. The removal of testosterone does not necessarily diminish libido, as libido is often more influenced by psychological well-being and hormone balance rather than testosterone alone. For patients experiencing dysphoria related to their genitals, orchiectomy often alleviates distress, making sexual experiences more positive and affirming [10].

### Hormonal deficiency and cognitive changes

#### Cognitive changes and hormonal deficiency

##### Impact on cognitive function

Androgen deprivation, whether through orchiectomy or chemical means, has been associated with cognitive changes. In a study by Yawson et al. (2021) using a rat model, androgen deprivation led to altered learning and cognitive behavior, characterized by decreased correct alternation and increased escape latency, indicating potential neurodegenerative changes in the hippocampus [11]. However, in human studies by Parikh et al (2019), no significant difference in the risk of dementia or Alzheimer’s disease was found between men undergoing androgen deprivation therapy (ADT) and those who had an orchiectomy [12].

#### Hormonal changes post-orchiectomy

##### Pituitary-Leydig cell axis

Orchiectomy can lead to significant hormonal changes, particularly affecting the pituitary-Leydig cell axis. According to Bandak et al (2011) and Wiechno et al (2017) After orchiectomy, there is often an increase in luteinizing hormone (LH) levels, indicating potential Leydig cell dysfunction. This hormonal imbalance may lead to hypogonadism, although the long-term need for androgen therapy remains uncertain [13]

##### Hormonal disorders

Men treated for testicular tumors often experience hormonal disorders, including changes in testosterone, estradiol, and LH levels. These changes are significant and can lead to testosterone deficiency, which is more pronounced after orchiectomy Wiechno et al (2017) [14]

## Factors influencing psychological outcomes

### Patient demographics

- In a study by booth et al (2019) Older patients tend to utilize mental health services (MHS) more frequently than younger patients following orchiectomy. At six months post-surgery, MHS usage was 15% for ages 16–29, 18% for ages 30–39, and 20% for those aged 40 and above. This suggests that older patients may experience or recognize psychological distress more acutely, or they may be more proactive in seeking mental health support compared to their younger counterparts [15].

### Social and cultural context

- Cultural stigma surrounding masculinity and bodily integrity may contribute to psychological distress; however, no studies have been identified on this topic. Therefore, further research is warranted, as preliminary intuition suggests its potential significance.

### Pre-existing mental health conditions

Patients with a history of mental illness, such as anxiety or depression, are more likely to experience adverse postoperative outcomes. A study by Paredes et al. (2020) found that Medicare beneficiaries with pre-existing mental illness had higher odds of surgical complications, extended hospital stays and increased 30-day readmission rates compared to those without mental illness. Additionally, these patients had a higher prevalence of suicidal ideation within the first-year post-surgery [16].

In the context of testicular cancer, Raphael et al. demonstrated that survivors who underwent orchiectomy were more likely to utilize mental health services both during the perioperative period and long-term post-treatment, especially if they had a history of mental health service use prior to surgery [2]. This suggests that pre-existing mental health conditions can exacerbate the psychological burden associated with cancer treatment and recovery. For patients undergoing orchiectomy for prostate cancer, Louda et al. reported that while mood disorders were less frequent post-surgery, there was a significant increase in sleep disorders during hospitalization and after discharge [5]. This indicates that the psychological impact of orchiectomy can vary, with some symptoms improving and others worsening. Overall, these findings underscore the importance of comprehensive preoperative mental health assessments and the integration of mental health services into the perioperative care plan for patients undergoing orchiectomy. This approach can help mitigate the adverse effects of pre-existing mental health conditions on surgical outcomes and improve overall patient care.

### Preventive measures and management strategies

#### Preventive measures

- **Preoperative Counselling**: Providing detailed information about the procedure, potential side effects, and expected outcomes can help mitigate anxiety and distress. This includes discussing alternatives to orchiectomy, such as androgen deprivation therapy (ADT) [17].
- **Psychosocial Support**: Engaging patients in psychosocial support services, including counselling and support groups, can help them cope with the emotional impact of orchiectomy. The American Psychosocial Oncology Society and the Association of Oncology Social Work recommend proactive identification and management of mental health issues [18].
- **Patient Satisfaction and Genital Perception**: Singh et al. (2021) suggested that Techniques like bilateral subcapsular orchiectomy (BSO) aim to preserve the perception of palpable testes, although satisfaction levels between BSO and bilateral extracapsular orchiectomy (BEO) are similar [19]

#### Treatment strategies

1. Stepped or Collaborative Care Models: These models involve regular screening for distress using validated tools such as the Patient Health Questionnaire (PHQ-9) for depression and the Generalized Anxiety Disorder (GAD-7) measure for anxiety. Patients are triaged based on their level of distress and provided with appropriate interventions, ranging from psychoeducation and supportive care to specialist mental health services for those with moderate-to-severe symptoms [17].
2. Regular Monitoring: Continuous assessment of psychological distress is crucial, especially in the first 24 months post-surgery. This includes monitoring for symptoms of depression, anxiety, and suicidal ideation, and adjusting care plans accordingly [18].
3. Specialist Referral: For patients with significant distress or preexisting mental health conditions, referral to a psychologist or psychiatrist is recommended. This ensures that patients receive specialized care tailored to their needs [20].
4. Cognitive Behavioural Therapy (CBT): CBT has shown efficacy in reducing depressive symptoms and anxiety, particularly in cancer survivors. It equips patients with strategies to challenge negative thought patterns and build resilience.
5. Supportive Psychotherapy: Providing a safe space for patients to express emotions can help normalize feelings of loss and uncertainty.
6. Peer Support Programs: Connecting patients with peer groups fosters a sense of community and shared understanding. Survivors and transgender individuals alike benefit from hearing others’ experiences, which normalizes emotional responses and offers practical coping strategies.

Implementing these strategies can help manage and reduce mental distress in patients undergoing orchiectomy, improving their overall quality of life and psychological well-being

### Future directions for research

1. **Personalized Mental Health Interventions**: More research is needed to tailor psychological care based on patient demographics.
2. **Cross-Cultural Studies**: Evaluating how different societies perceive and manage the psychological consequences of orchiectomy.
3. **Longitudinal Assessments**: Long-term follow-up studies are needed to map the evolving mental health trajectories of post-orchiectomy patients.

### Limitations

- Variability in psychological assessment tools across studies.
- Lack of high-quality randomized controlled trials.
- Cultural differences may limit generalizability.

## Conclusion

**Figure 2.**
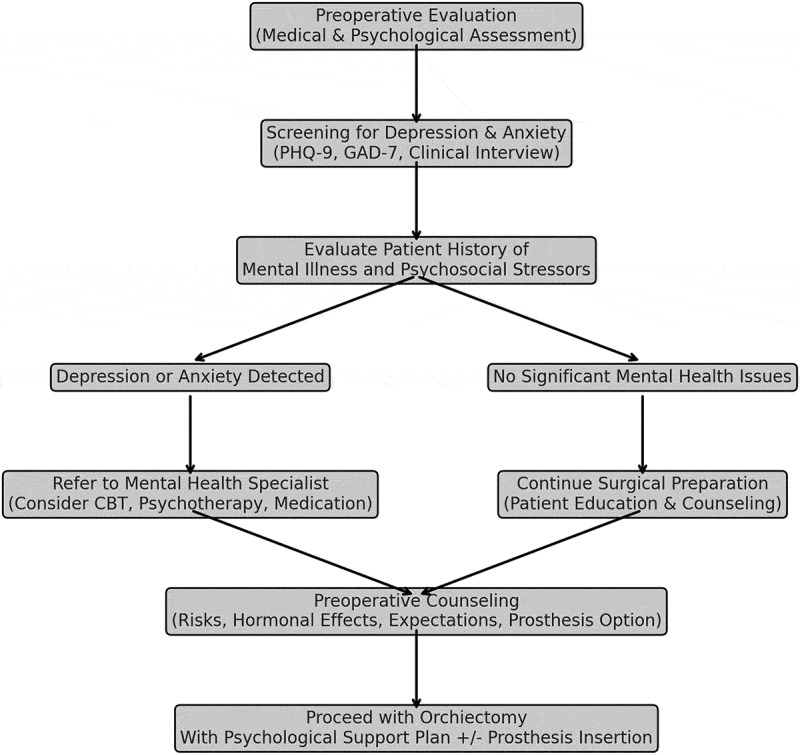
Pre-operative evaluation and counselling outcomes.

Orchiectomy is a medically necessary but psychologically complex procedure. While it effectively addresses underlying health conditions, the emotional consequences require a multidisciplinary approach. Pre-operative evaluation and counselling (Figure 2), hormonal therapy, and targeted psychological interventions are crucial for mitigating adverse effects. Future research should prioritize longitudinal evaluations and culturally sensitive interventions to enhance patient outcomes.
